# Recurrent Episodes of Paroxysmal Supraventricular Tachycardia Triggered by Dyspepsia: A Rare Case of Gastrocardiac Syndrome

**DOI:** 10.7759/cureus.17966

**Published:** 2021-09-14

**Authors:** Khadija Qureshi, Nauman Naeem, Shoaib Saleem, Maida S Chaudhry, Fajar Pasha

**Affiliations:** 1 Internal Medicine, Bucks County Kidney Specialists, Langhorne, USA; 2 Internal Medicine, Allama Iqbal Memorial Hospital, Sialkot, PAK; 3 Medicine, District Headquarter Hospital, Kotli, PAK; 4 Internal Medicine, DHR Health Institute for Research and Development, Edinburg, USA; 5 Internal Medicine, Rawalpindi Medical University, Rawalpindi, PAK; 6 Internal Medicine, Holy Family Hospital, Rawalpindi, PAK

**Keywords:** gastrocardiac syndrome, vagal response, supraventricular tachycardia, dyspepsia, abdominal bloating

## Abstract

Supraventricular tachycardia (SVT) refers to the narrow complex tachycardia originating at or above the bundle of His. Several risk factors are associated with the development and recurrence of SVT, but its association with gastric problems, especially dyspepsia, is relatively rare. We report the case of a 54-year-old female who presented to the emergency room (ER) with palpitations, which were diagnosed as an episode of paroxysmal supraventricular tachycardia (PSVT). She had a history of PSVT in the past, along with hypertension and dyspepsia. After thorough history and examination, dyspepsia was identified as the common trigger of her PSVT episodes, pointing towards the likelihood of gastrocardiac symptoms. Therefore, an appropriate regimen of beta-blockers, proton pump inhibitors (PPIs), and anti-foaming agents (simethicone) was prescribed to manage her symptoms with the plan to perform a catheter ablation later.

## Introduction

Supraventricular tachycardia (SVT) is a tachyarrhythmia originating from the cardiac tissue at the level of the bundle of His [a specialized tissue transmitting electrical impulses from the atrioventricular (AV) node to the Purkinje fibers] or above [1]. Tachycardia is defined as an atrial and/or ventricular rate of >100 beats per minute (bpm). SVT constitutes a heterogeneous group of arrhythmias such as atrial fibrillation, atrial flutter, atrial tachycardia, AV nodal re-entrant tachycardia (AVNRT), AV re-entrant tachycardia (AVRT), and permanent junctional reciprocating tachycardia (PJRT) [2]. The prevalence of SVT is 2.25 per 1,000 people with a female predominance of 2:1 across all age groups. The risk of increasing patient morbidity with paroxysmal supraventricular tachycardia (PSVT) is directly proportional to the frequency of episodes, and it can be life-threatening in patients with atrial fibrillation and ventricular pre-excitation [1]. Dyspepsia is a symptom complex including epigastric pain, bloating, early satiety, postprandial fullness, epigastric burning, belching, bloating, nausea, and vomiting. It is highly prevalent and mostly chronic and recurrent in nature. It is caused by several mechanisms, including delayed gastric emptying, impaired gastric accommodation to a meal, hypersensitivity to gastric distention, *Helicobacter pylori (H. pylori)* infection, altered response to duodenal lipids or acids, abnormal duodenojejunal motility, abnormalities of gastric electrical rhythm, and autonomic nervous system dysfunction. Its management includes patient reassurance and education, lifestyle and dietary modifications, proton pump inhibitors (PPIs) (e.g., omeprazole, pantoprazole), H2 receptor blockers (e.g., famotidine, ranitidine), prokinetic agents (e.g., metoclopramide, domperidone, and cisapride), eradication of *H. pylori*, and tricyclic antidepressants (e.g., amitriptyline) [3]. Gastrocardiac syndrome (also known as gastric-cardia syndrome or Roemheld syndrome) involves a complex of symptoms where issues with the gut are found to be associated with cardiac symptoms like shortness of breath, chest pain, nausea, palpitations, skipped beats, and arrhythmias. It could be secondary to mechanical, hormonal, chemotoxic, and structural defects (e.g., hiatal hernia) in the gastrointestinal system. This symptom complex arises due to the intricate relationship of the gut and heart with the nervous system, with the vagal nerve being the main link between the two [4]. The treatment primarily focuses on controlling heart rate along with reducing dyspeptic and other gastrointestinal concerns [5].

## Case presentation

**Figure 1:**
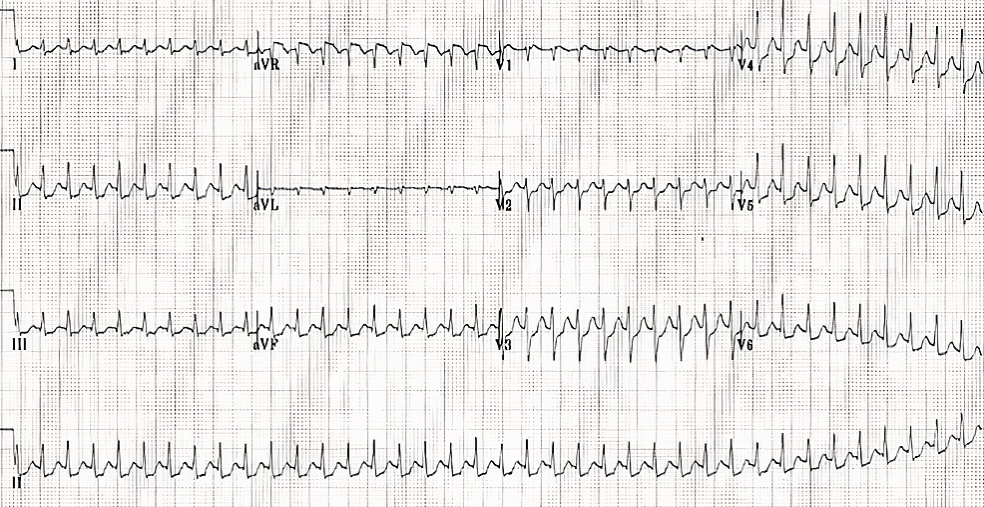
12-lead EKG showing narrow complex supraventricular tachycardia with a heart rate of 220 beats per minute EKG: electrocardiogram

A 54-year-old female patient presented to the emergency room (ER) with the chief complaint of a rapid heart rate and an uncomfortable feeling of being aware of her heartbeat. She had a long-standing history of hypertension for 20 years treated with losartan and hydrochlorothiazide (50/12.5 mg once daily), dyspepsia for almost 15 years managed with omeprazole (20 mg once daily), and a relatively recent past medical history of PSVT managed with metoprolol tartrate (25 mg twice daily). Her vitals showed a heart rate of 220 bpm with a blood pressure of 160/98 mmHg. She was saturating at 96% on room air without any dyspnea or chest pain. Valsalva maneuver and carotid massage did not resolve her symptoms. Electrocardiogram (EKG) strips were immediately recorded, which showed a narrow-complex tachycardia with a heart rate of 220 bpm, without clear P-waves, pointing towards PSVT (Figure 1). The initial EKG recording during the paroxysmal episode of SVT is shown below.

**Figure 2:**
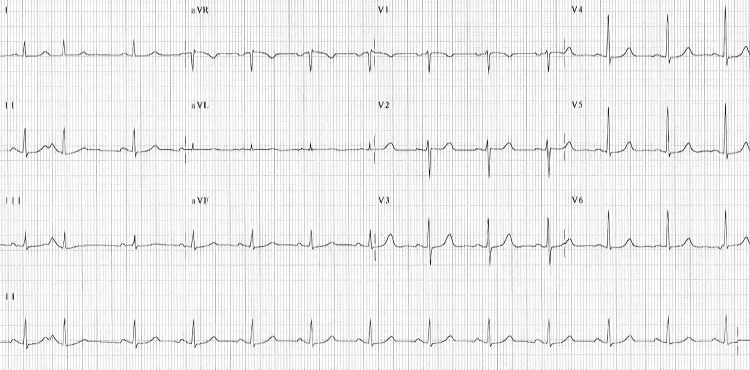
12-lead EKG showing normal sinus rhythm with a heart rate of 85 beats per minute EKG: electrocardiogram

**Figure 3:**
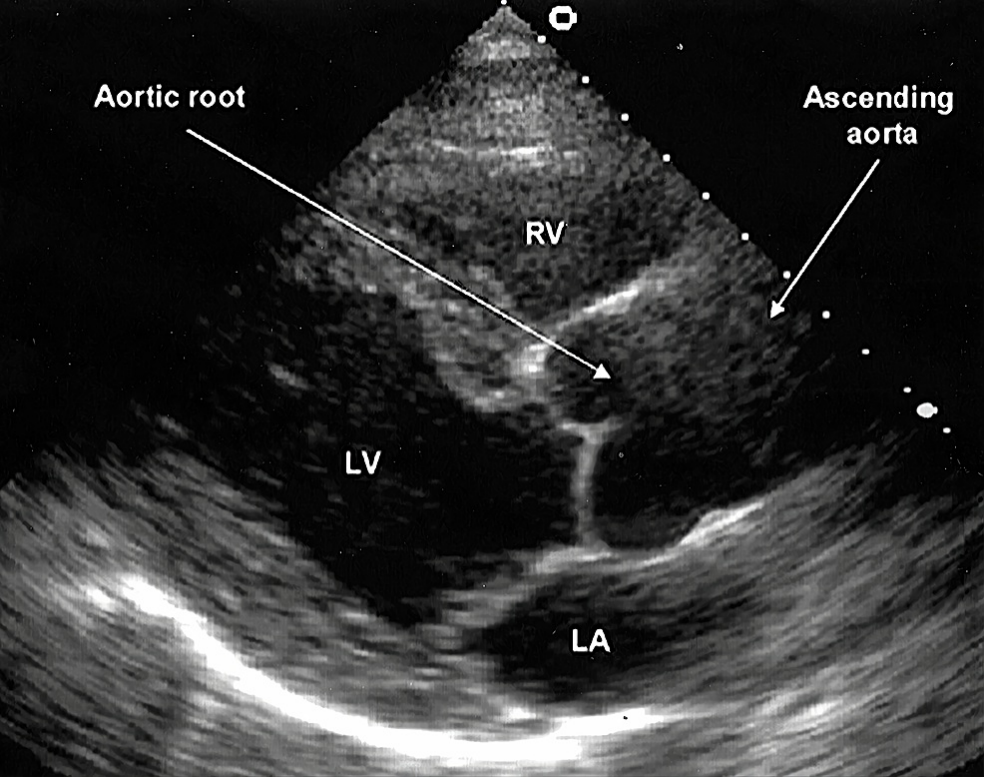
Transthoracic echocardiogram showing normal findings

Six milligrams of adenosine were administered to the patient via a catheter in the peripheral vein of her right hand, immediately followed by a 10 mL rapid saline flush. Adenosine brought her heart rate down to normal sinus rhythm (Figure 2). The patient was started on telemetry with continuous heart rate monitoring until the readings were stable around 80 bpm. Initial blood workup, including complete blood count, basic metabolic profile, cardiac enzymes (troponins and brain natriuretic peptide), and thyroid function tests, was within normal ranges. Troponin levels were monitored at zero, one, and four hours, which stayed within the normal range. Chest X-ray showed no abnormalities. The transthoracic echocardiogram (TTE) revealed normal right and left atrial chambers, normal right and left ventricular sizes, no valvular or wall defects, no cardiomegaly, and an average ejection fraction of 60-65% (Figure 3).

The patient stabilized but reported experiencing belching and bloating. She had been having uncomfortable bloating, abdominal distension, and nausea continuously for the past 20 hours. Her bowel movements were regular without any constipation or diarrhea, and she was passing flatus. The patient had taken her home medication of omeprazole 20 mg twice within the last 20 hours, but her dyspeptic symptoms continued without much relief. She reported a frequent occurrence of palpitations with her gastrointestinal symptoms. She had been previously admitted to the ER three times within the last year for episodes of PSVT. All of her PSVT episodes had been preceded by severe dyspepsia. A thorough gastrointestinal workup was performed, revealing no abnormalities. Abdominal X-ray and ultrasonography were normal with no evidence of gallstones or anatomical defects. Due to the long-standing nature of her dyspepsia, an endoscopy was scheduled, which revealed no defects or malformations. After an in-depth gastrointestinal investigation, a diagnosis of functional dyspepsia was established. The episode of SVT was thought to be linked to dyspepsia. The patient was prescribed omeprazole 40 mg daily and instructed to continue taking other home medications, including metoprolol tartrate (25 mg twice daily). Catheter ablation was discussed in detail with the patient, but she refused to undergo any invasive procedures and opted for medical management alone. In addition, she was thoroughly counseled regarding dietary and lifestyle modifications like having small frequent meals, limiting gluten and dairy products, decreasing fatty foods, avoiding spices and coffee, and exercising regularly. At the follow-up appointment four weeks later, she reported an improvement in her dyspepsia. She had recorded her blood pressure and heart rate every day with a home blood pressure machine, both of which had stayed within the normal range. The palpitations resolved with the reduction in her dyspepsia, indicating a relationship between the cardiac and gastric systems in her case.

## Discussion

SVTs are defined as narrow complex tachycardias. A collective data of the EKGs and cardiac electrophysiology studies have shown that 80% of SVTs are either AVNRT or AVRT cases, with up to 60% being AVNRT. AVNRT can occur due to the re-entrant electric circuits in the heart, triggered by excess catecholaminergic states, e.g., emotional strain, physical activity, and other stresses. The EKG findings in AVNRT usually do not easily show the P-waves during the paroxysmal episodes. This is due to the simultaneous activation of the atria and ventricles, and the heart rate being too rapid to allow for the proper atrial filling. EKG also shows a pseudo-R-wave in lead V1 or a pseudo S deflection in the inferior leads. A pseudo S deflection has a higher specificity and positive predictive value for AVNRT [6]. Abnormalities within the autonomic nervous system have considerable importance in the pathophysiology of functional dyspepsia. A dysregulated vagus nerve stimulation is considered a possible cause of impaired accommodation to a meal and antral hypomotility [3]. The vagus nerve (CN X) is the 10th cranial nerve that originates from the medullary nuclei in the brain and leaves the skull through the jugular foramen, providing numerous vagaries to the neck, throat, chest, and abdominal regions. As the vagus nerve enters the abdominal cavity through the esophageal hiatus, it splits into an anterior trunk and a posterior trunk. The anterior trunk is mainly responsible for gastrointestinal parasympathetic innervation to the antral and distal portions of the stomach, the pylorus, the biliary apparatus, and the gallbladder [7]. More than 75% of dyspeptic patients report aggravation of symptoms (in 45-90 minutes) after the consumption of a standardized 250-kilocalorie (kcal) meal. Dyspepsia leads to postprandial fullness, bloating, and stomach distension. One major reason for bloating is the unbalanced bacterial flora in the gut, which leads to excessive fermentation of the food and gas formation. The gastrointestinal accumulation of air hampers the normal digestion and motility of food, distends the stomach and intestines, exerts pressure on surrounding organs, and elevates the diaphragm. All these mechanisms cause stretching of the vagus nerve. The over-activation of the vagus nerve is responsible for an exaggerated vagal response, which is not just limited to the gastrointestinal region but also affects other organ systems, mainly the heart [3]. The vagus nerve has its visceral vagaries supplying the cardiac tissues, including the atria, ventricles, cardiac fat pads, and sinus and AV nodes. AV nodal hyper-polarization predominantly mediated by the left vagus nerve slows down the action potentials in the nodal circuits. The parasympathetic effects on heart rate and AV nodal conduction appear modulated generally via M2 receptors, which markedly slow the heart rate [mediated in part by G-protein inwardly rectifying potassium (GIRK) channels]. Therefore, the higher the vagus nerve activity, the slower the heart rate [8]. Multiple studies have demonstrated the critical role of the cardiac autonomic nervous system in cardiac arrhythmogenesis. Furthermore, the atrial effective refractory period is reduced by vagal stimulation, thereby predisposing the patients to paroxysmal atrial arrhythmias [9].

Up to 80% of SVT cases involve the AV node (AVNRT/AVRT). AVNRT is characterized by dual pathways in the AV node, one slow and one fast pathway. There is unbalanced refractoriness of the two nodal pathways: the fast pathway having a relatively long refractory period, while the slow pathway often has a shorter refractory period. During sinus rhythm, nodal conduction usually occurs through the fast pathway. The exaggerated vagal response caused by abdominal distension secondary to dyspepsia inhibits both the slow and fast pathways. Since the refractory period of the slow pathways is shorter than the fast pathways, a premature atrial beat may find this pathway available and cross the AV node through it. The result is a sudden prolongation of the AV conduction time. When the impulse reaches the common end, it excites the fast pathway as well and triggers a re-entrant tachyarrhythmia [10].

There have been documented cases of patients presenting for arrhythmic disorders secondary to gastrointestinal defects. For example, a 75-year-old female patient experienced severe recurrent bradycardia with each episode of abdominal bloating. Her bradycardia resolved with the resolution of gastric distension [11]. Another case involving a 77-year-old female patient with recurrent atrial fibrillation was diagnosed to be secondary to paraesophageal hernia, and the hernia repair led to the resolution of her arrhythmic episodes [12]. A meta-analysis of seven studies endorsed the association of atrial fibrillation (a form of tachyarrhythmia) with gastroesophageal reflux disease (GERD) [13]. An extensive study has shown that GERD was independently associated with an increased risk of future atrial fibrillation in a nationwide population-based cohort [14]. Another study that evaluated patients with idiopathic supraventricular cardiac dysrhythmias identified a subgroup of dysrhythmic patients in whom the esophageal acid stimulus elicited cardiac autonomic reflexes. In these patients, acid suppression resulted in the improvement of GERD and cardiac symptoms [15]. This data could be logically used as evidence of the interrelationship between gastric and cardiac systems. The analysis so far suggests that gastrointestinal disorders can act as risk factors or causative elements of cardiac arrhythmias. However, more studies are needed to explore the complex nature of gastrocardiac syndrome.

## Conclusions

Physicians should be mindful of the relationship between gastrointestinal dysfunction and cardiac arrhythmias. The association between these two organ systems is often overlooked, and the diagnosis of gastrocardiac syndrome is relatively rare. However, this diagnostic approach can tremendously alter the treatment regimen and management of dysrhythmic patients. Physicians should suspect the probability of unidentified and/or untreated gastrointestinal factors in patients with arrhythmic disorders. A detailed history of dietary and bowel habits should be obtained from all cardiac patients. If a gastric dysfunction or defect is suspected, the care team consisting of cardiologists, gastroenterologists, and dieticians should be coordinated, ensuring a combined effort to best address the underlying problems and comorbidities in the patients.
